# Improving performance in SNN-based deep reinforcement learning via transition-aware embeddings

**DOI:** 10.3389/fnbot.2026.1929177

**Published:** 2026-10-05

**Authors:** Hiroto Takigasaki, Takahiro Iwata, Seankein Yoshioka, Daisuke Miki

**Affiliations:** Graduate School of Information and Computer Science, Chiba Institute of Technology, Narashino, Chiba, Japan

**Keywords:** continuous control, deep reinforcement learning (DRL), reinforcement learning, representation learning, spiking neural network (SNN)

## Abstract

Deep Reinforcement Learning (DRL), which integrates reinforcement learning with deep neural networks (DNNs), has been extensively researched across diverse domains. Robotics, in particular, has seen significant advancements, as DRL enables agents to extract meaningful features from high-dimensional observations and make precise decisions within complex environments. Furthermore, in real-world control tasks, achieving high performance within a limited number of interactions is required, particularly in complex and unknown environments. In addition, since real-world trials are constrained by cost and safety considerations, designing DRL algorithms that enable high sample efficiency and stable decision-making is of critical importance. Spiking neural networks (SNNs), inspired by biological neural systems, have recently attracted increasing attention due to the binary and event-driven nature of their information processing. Motivated by these properties, there has been growing interest in integrating SNNs into DRL. Nevertheless, SNN-based DRL faces challenges in learning performance. To address this issue, this study introduces embeddings learned from environmental dynamics into SNN-based DRL. Experimental evaluations conducted on four continuous control tasks from OpenAI Gym demonstrate that the proposed method outperforms conventional SNN-based DRL methods, both in terms of maximum average rewards and accelerated reward acquisition during the early stages of learning. These results establish that leveraging transition-aware embeddings effectively enhances both performance and sample efficiency in SNN-based DRL.

## 1 Introduction

Reinforcement learning (RL) is a machine learning framework wherein an agent learns to make optimal decisions through interactions with an environment to maximize the expected cumulative reward. Deep reinforcement learning (DRL), which uses deep neural networks (DNNs) as function approximators, is employed to address complex problems involving high-dimensional or continuous state spaces. Owing to its high representational capacity, DRL has achieved remarkable success in recent years across various fields, including games (Mnih et al., 2015; Hafner et al., 2024), robotics (Haarnoja et al., 2018; Tang et al., 2024), autonomous driving (Kiran et al., 2021), natural language processing (He et al., 2016), and healthcare (Raghu et al., 2017). Among these domains, DRL has attracted particular interest in robotics as a decision-making method that enables flexible action selection in dynamic, changing environments. However, deploying autonomous mobile robots in real-world environments necessitates large-scale neural networks to handle high-dimensional observation and action spaces, which in turn increases energy consumption. Because autonomous mobile robots operate with limited onboard resources, energy-efficient decision-making methods are essential.

Against this background, spiking neural networks (SNNs) have emerged as a compelling alternative to conventional artificial neural networks (ANNs). By transmitting information through discrete spikes, SNNs have been reported to enable low-power computation on neuromorphic devices. In recent years, the integration of SNNs with DRL has been recognized as an energy-efficient solution for robotic control (Tang et al., 2020; Naya et al., 2021). Tang et al. proposed a representative method that replaces the actor with an SNN in actor-critic based DRL, referred to as the Population-Coded Spiking Actor Network (PopSAN) (Tang et al., 2021). While PopSAN has been reported to be significantly more energy-efficient than deep actor networks in continuous control tasks, challenges remain in improving the learning performance of SNN-based DRL methods (Oikonomou et al., 2023; Zhang et al., 2025). Potential factors contributing to this include information loss caused by spike encoding (Guo et al., 2022a) and representational constraints arising from spike-based computation (Guo et al., 2022b; Lee et al., 2020). In SNNs, information is propagated through spike trains rather than continuous-valued activations. Therefore, compared with an ANN-based actor, a spiking actor network is at a disadvantage in forming continuous-valued features that are useful for policy learning within the network.

Representation learning offers a promising approach to improving the inputs used for policy learning and action-value estimation. For example, in representation learning for RL, Fujimoto et al. proposed State-Action Learned Embedding (SALE) (Fujimoto et al., 2023), a method that jointly learns embeddings for both state and action by modeling the dynamics of the environment in a latent space. By leveraging these learned embeddings in both the policy and the action-value function (Q-function), improvements have been reported in both final performance and sample efficiency during the early stages of training. In this study, we propose a framework that integrates SALE into a hybrid architecture comprising a spiking actor network and critic network. Our contribution lies in this integration of transition-based representation learning with spiking policy learning and its empirical evaluation on continuous control tasks. We examine the contribution of embedding inputs and the influence of embedding dimensionality on learning performance.

The remainder of this paper is organized as follows. Section 2 reviews related work, and Section 3 describes the proposed method and its foundations. Section 4 presents the experimental setup and results. Section 5 discusses the findings, and Section 6 concludes the paper and outlines future work.

## 2 Related work

### 2.1 RL in SNN

SNNs have been increasingly applied to DRL, motivated by their biological plausibility and the potential for significantly improved energy efficiency when deployed on neuromorphic chips. Prior research in this area can be broadly categorized into three primary approaches.

#### 2.1.1 ANN to SNN conversion

This approach enables SNN-based inference by transferring the weights of a pretrained ANN to an SNN. Patel proposed a method to convert a well-trained Deep Q-Network (DQN) (Mnih et al., 2015) into an SNN, reporting improvements in both energy efficiency and robustness (Patel et al., 2019). However, it is generally observed that the converted SNN does not outperform the original ANN counterpart (Tan et al., 2020).

#### 2.1.2 Direct training of SNNs

This approach allows for the direct training of SNNs without requiring conversion from ANNs. Surrogate gradients are typically applied to the non-differentiable spike firing function, enabling end-to-end optimization. Liu et al. reported that a directly trained Deep Spiking Q-Network outperforms both ANN-based DQN and converted SNN-DQNs on average across Atari benchmarks, while also achieving enhanced stability and robustness (Liu et al., 2023). Furthermore, Chen et al. proposed “spiking Q-learning,” wherein Q-values are represented by membrane potentials (Chen et al., 2024). By employing surrogate gradient learning, their method improves training stability and robustness to perturbations, while showing significant promise regarding energy efficiency. However, these methods are primarily designed for discrete action spaces, and challenges remain in extending them effectively to continuous control tasks.

#### 2.1.3 Hybrid frameworks with spiking actor networks

For tasks involving continuous action spaces, hybrid frameworks have been proposed that combine a spiking actor network with a deep critic network. As a prominent example, Tang et al. proposed PopSAN based on population coding, in which continuous values are represented by the firing rates of multiple neurons (Tang et al., 2021). PopSAN enhanced the representational capacity of the network, enabling effective learning in continuous control environments. Building upon this population-coding framework, Zhang et al. proposed a multiscale dynamic coding-improved spiking actor network (MDC-SAN) (Zhang et al., 2022), which combines population coding at the network scale with dynamic neurons incorporating second-order neuronal dynamics at the neuron scale. This design aims to improve spatiotemporal state representations for continuous control. Building on these spiking actor approaches, Iwata et al. proposed an SNN-based DRL method featuring a non-recursive SNN structure to eliminate output delays (Iwata et al., 2024). Furthermore, to address the issue of “stale” membrane potentials in the replay buffer, they introduced the “burn-in” strategy proposed in recurrent replay distributed DQN (R2D2) (Kapturowski et al., 2019), achieving improved Q-function estimation accuracy.

### 2.2 Representation learning

Representation learning is a field of machine learning that aims to automatically extract meaningful features from raw data and acquire embeddings that are useful during the learning process. In DRL, representation learning that extracts useful features from high-dimensional observations, such as images, is considered vital for improving performance and sample efficiency. More recently, the effectiveness of representation learning has also been demonstrated for tasks employing low-dimensional state observations. Ota et al. proposed an online feature extraction network (OFENet) that learns high-dimensional state and state-action representations through an auxiliary task of predicting the next observation (Ota et al., 2020). Fujimoto et al. proposed State-Action Learned Embedding (SALE), which instead learns a state-action embedding to predict the next-state embedding (Fujimoto et al., 2023). By modeling environmental dynamics, SALE provides features that capture the interaction between state and action, which can facilitate learning of the policy and Q-function. Both methods therefore use environmental transitions to learn representations; a key distinction is whether the prediction target is an observation or a learned embedding. These studies demonstrate the effectiveness of representation learning in ANN-based DRL. However, the effectiveness of these representations in SNN-based DRL, where computation is performed using binary spike signals, requires further investigation.

## 3 Methods

### 3.1 DRL

RL is a framework in which an agent learns a policy π(*a*∣*s*) that maximizes the expected reward through interactions with the environment. At each time step, the agent observes the current state *s*, selects an action *a* according to the policy, and receives a reward *r*. By repeating this process, the agent aims to acquire an optimal policy that maximizes the return. DRL integrates DNNs into this framework, enabling the approximation of policies π(*a*∣*s*) and Q-functions *Q*(*s, a*) in high-dimensional state and action spaces.

For tasks requiring continuous action selection, actor-critic algorithms (Barto et al., 1983), which employ separate networks for policy and value estimation, are widely used. Among these, the deep deterministic policy gradient (DDPG) (Lillicrap et al., 2016) is a deterministic actor-critic algorithm comprising an actor network that produces continuous actions and a critic network that estimates the corresponding action-value. To address the overestimation bias often found in DDPG, twin delayed DDPG (TD3) was proposed (Fujimoto et al., 2018). TD3 improves learning stability through three key techniques: clipped double Q-learning, delayed policy updates, and target policy smoothing. By mitigating overestimation bias, TD3 has become one of the most widely used DRL algorithms for continuous control.

### 3.2 SNN

As ANNs become increasingly deep and large-scale, their computational cost and power consumption have become critical issues. SNNs, inspired by biological systems, communicate information through discrete spike trains. Owing to their event-driven nature, SNNs demonstrate high energy efficiency on neuromorphic hardware platforms such as Intel's Loihi (Davies et al., 2018) and IBM's TrueNorth (Merolla et al., 2014).

#### 3.2.1 Neuron model

A diverse array of neuron models has been developed for neural networks, ranging from the biophysically detailed Hodgkin–Huxley model (Hodgkin and Huxley, 1952) to artificial neuron models, such as the perceptron (Rosenblatt, 1958), that are widely employed in deep learning. The Hodgkin–Huxley model provides high biological fidelity and supports precise electrophysiological computations; however, it is notably computationally expensive. By contrast, artificial neuron models prioritize computational efficiency by adopting simplified mathematical architectures, which may lead to significant discrepancies from actual electrophysiological behavior. To strike a balance between biological realism and computational tractability, the leaky integrate-and-fire (LIF) model (Gerstner and Kistler, 2002) was introduced as a streamlined spiking neuron model. The LIF model is extensively used as a standard for spiking neurons and is described as follows:


$$\begin{array}{l} \tau \frac{dV\left(t\right)}{dt}=\left(-V\left(t\right)+V_{\text{rest}}\right)+I\left(t\right) \end{array}$$ (1)

where *V*(*t*) denotes the membrane potential, *V*_rest_ represents the resting potential, *I*(*t*) is the input current, and τ is the membrane time constant. When the membrane potential reaches a predefined threshold *V*_th_, the neuron emits a spike, after which the potential is reset to *V*_reset_. In this study, the LIF model is adopted as the fundamental neuron model for the SNN.

#### 3.2.2 Spiking actor network

The spiking actor network is an architecture that replaces the traditional actor network in actor-critic algorithms with an SNN to reduce power consumption during the inference phase. A typical spiking actor network architecture comprises a spike encoder that converts continuous environmental observations into discrete spike trains, a multilayer SNN that processes these encoded signals, and a decoder that transforms the resulting spiking outputs back into continuous-valued actions. Various spike encoding schemes, including population and rate coding, are employed to represent continuous values as temporal spike trains. It is important to note that inherent information loss occurs during this conversion process from continuous signals into discrete spikes.

### 3.3 Proposed method

In this study, we adopt the TD3 algorithm (Fujimoto et al., 2018), in which the actor is implemented as a spiking actor network. Furthermore, we introduce SALE, a representation learning method, to obtain and utilize embeddings that capture the transition dynamics of the environment. In the spiking actor network, representational constraints arising from spike-based computation make it difficult to form useful features for policy learning. Providing the state embedding learned by the SALE encoders before spike encoding is intended to support policy learning without relying solely on feature formation within the SNN. For the critic networks, following Fujimoto et al., we introduce state and state-action embeddings to support Q-function approximation using features that capture environmental transition dynamics. Since the actor is updated using the critics' action-value estimates, supporting the critics is also expected to benefit policy learning. Additionally, to improve the stability of evaluation results, we employ the policy checkpoint mechanism proposed by Fujimoto et al. (2023). The training process for SALE and its integration into the critic network are illustrated in Figure 1. The architecture of the spiking actor network is depicted in Figure 2. Finally, the overall training procedure is summarized in Algorithm 1. Each component is detailed below.

**Figure 1:**
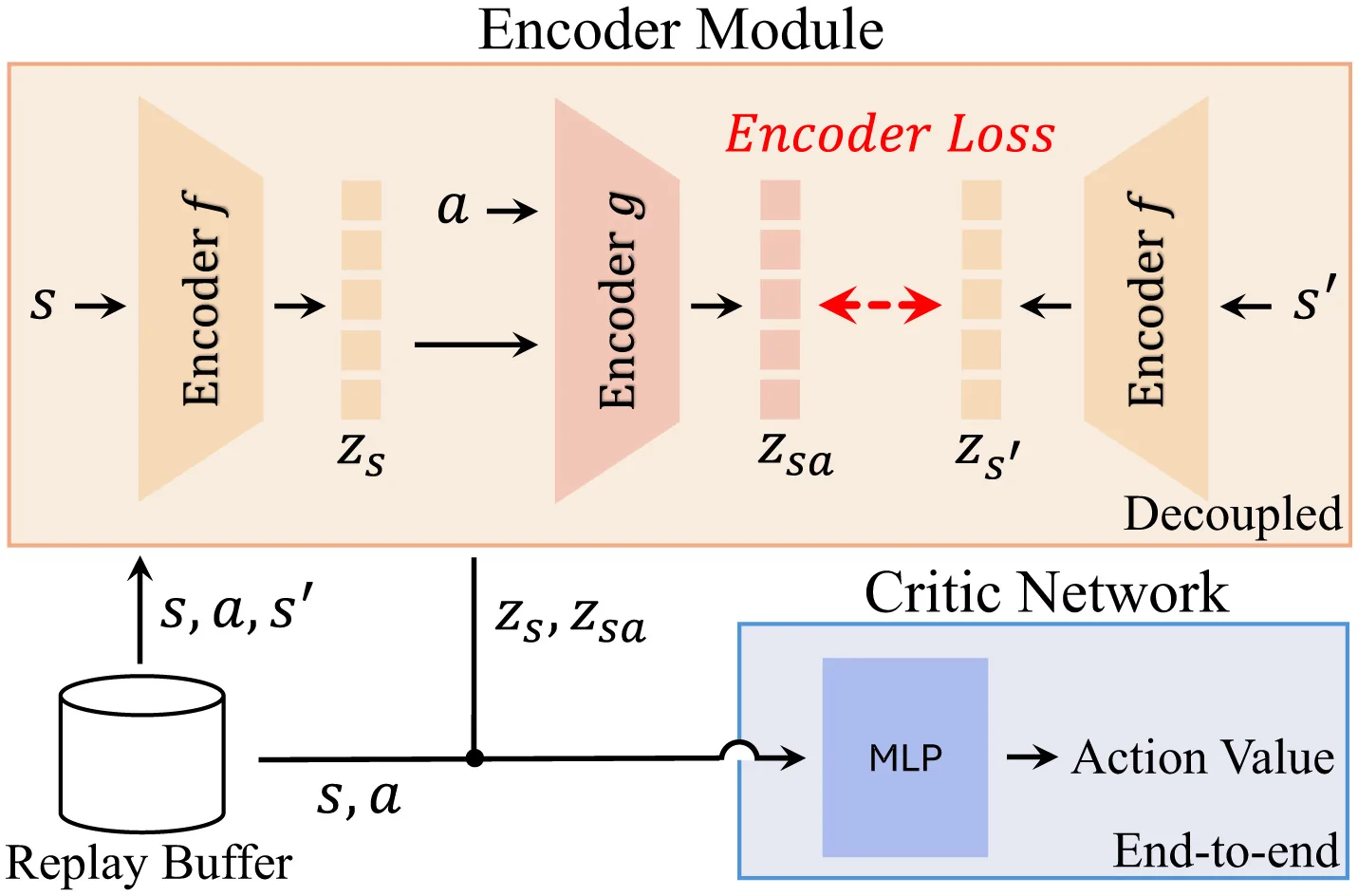
Structure of the encoder module for learning transition-aware embeddings and its integration into the critic network. The encoder learns the representations *z*_*sa*_ and $z_{s^{\prime }}$ from the transition dynamics of the environment. The learned embeddings (*z*_*s*_, *z*_*sa*_) are subsequently provided to the critic to facilitate action-value estimation.

**Figure 2:**
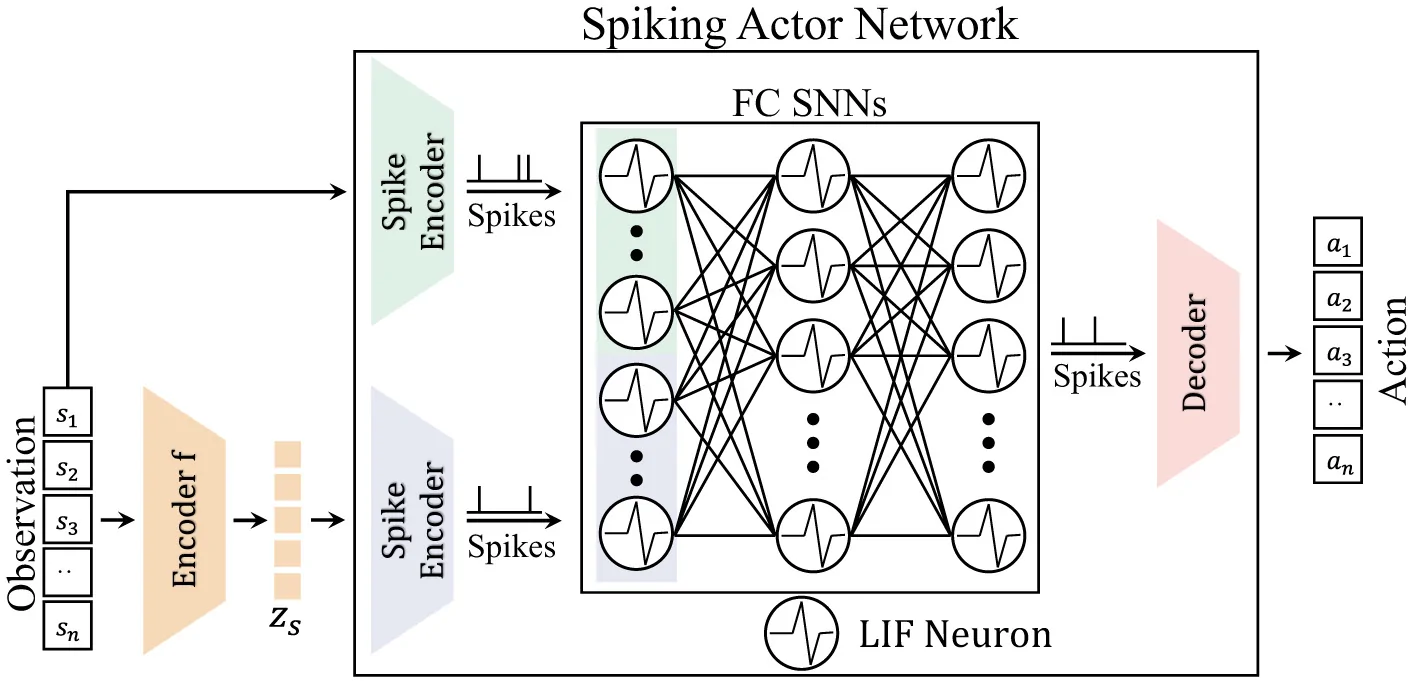
Architecture of the spiking actor network. The raw observation *s* and its corresponding state embedding *z*_*s*_ are converted into discrete spike trains by specialized spike encoders. These spike-based representations are processed through fully connected SNN layers, and continuous actions are generated by the decoder.

Algorithm 1 **Training Procedure of Spiking TD3 with Transition-Aware Embeddings**

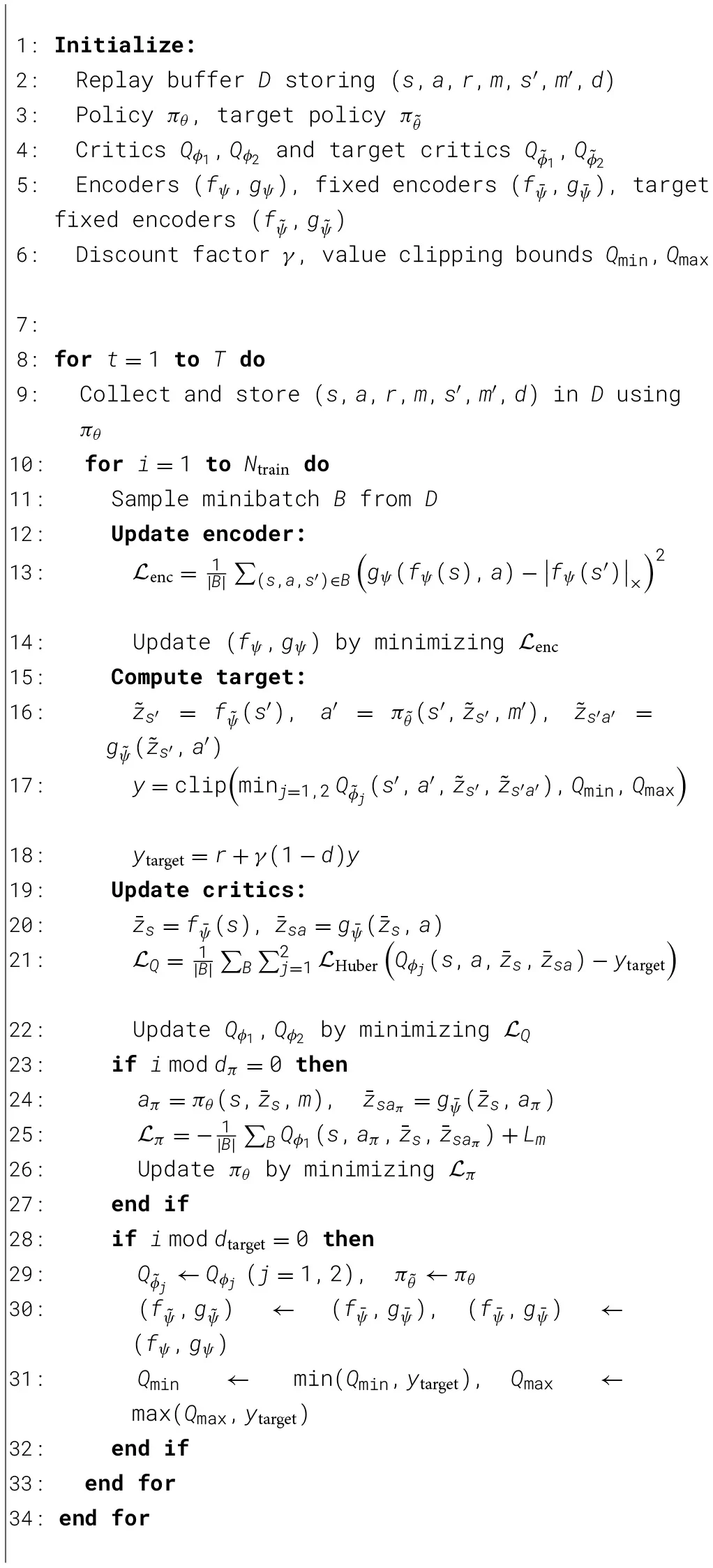

#### 3.3.1 SALE

We employed SALE, proposed by Fujimoto et al. (2023), as a representation learning approach to model the interaction between state and action. SALE is designed to acquire embeddings reflecting both the inherent structure of the observation space and the transition dynamics of the environment. By using these embeddings as inputs for the Q-function and the policy, SALE enhances both the final performance and the early stages of learning. As shown in Figure 1, the state *s* is first processed by an encoder *f* to obtain the state embedding *z*_*s*_. Subsequently, the state embedding and action serve as inputs to another encoder *g*, which produces the state-action embedding *z*_*sa*_. These encoders (*f, g*) are jointly trained to minimize the mean squared error (MSE) between the state-action embedding *z*_*sa*_ and the next-state embedding $z_{s}^{\prime }$. This encoder loss is defined as follows:


$$\begin{array}{l} L\left(f,g\right)={\left(z_{sa}-{\left|z_{s^{\prime }}\right|}_{\times }\right)}^{2}, \end{array}$$ (2)

where |·|_×_ denotes the stop-gradient operation. The encoders (*f, g*) are trained by minimizing the encoder loss in Equation 2, separately from the policy and Q-function losses. These latter losses do not update the encoder parameters. This design ensures the encoders acquire embeddings that reflect environmental dynamics independently of rewards or specific policies. The default embedding dimensions are set to *d*_*z*_*s*__, *d*_*z*_*sa*__ = 64, and their effects are analyzed through additional experiments.

#### 3.3.2 Actor network

In the proposed method, the actor is implemented as a spiking actor network based on SNNs. This network comprises a spike encoder, fully connected SNNs (FC-SNNs), and a decoder. As described in Algorithm 2, the spike encoder receives the raw continuous state *s* from the environment and the state embedding *z*_*s*_ from encoder *f*, converting them into spike trains. These are then propagated through a three-layer FC-SNN. Finally, as shown in Algorithm 3, the decoder transforms the generated spike trains into continuous-valued actions, which serve as the agent's control signals. The population dimension *d*_pop_ used in both the spike encoder and decoder is set to 32. The learnable parameters of the spike encoder and decoder are jointly optimized with the FC-SNN by minimizing the policy loss. Additionally, to suppress excessive spiking activity, we introduce an auxiliary membrane-potential regularization term *L*_*m*_, which penalizes the squared magnitude of the membrane potentials averaged over all neurons. This term is added to the policy loss, encouraging smaller membrane potentials and thereby reducing unnecessary spike generation.

Algorithm 2 **Spike Encoder**

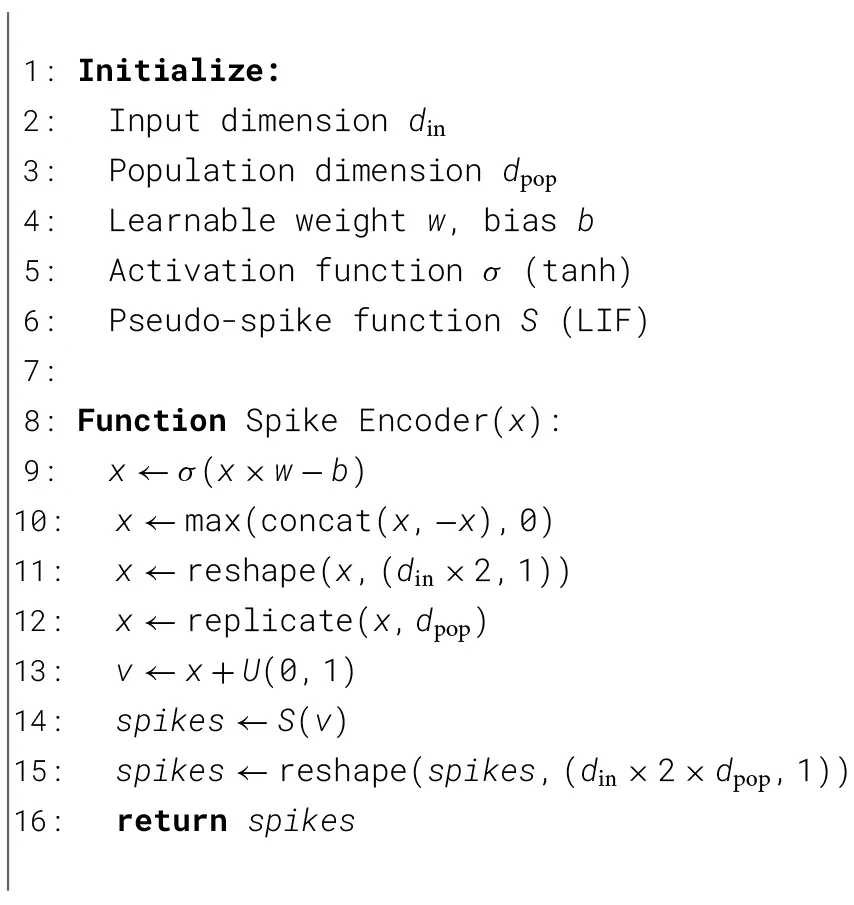

Algorithm 3 **Decoder**

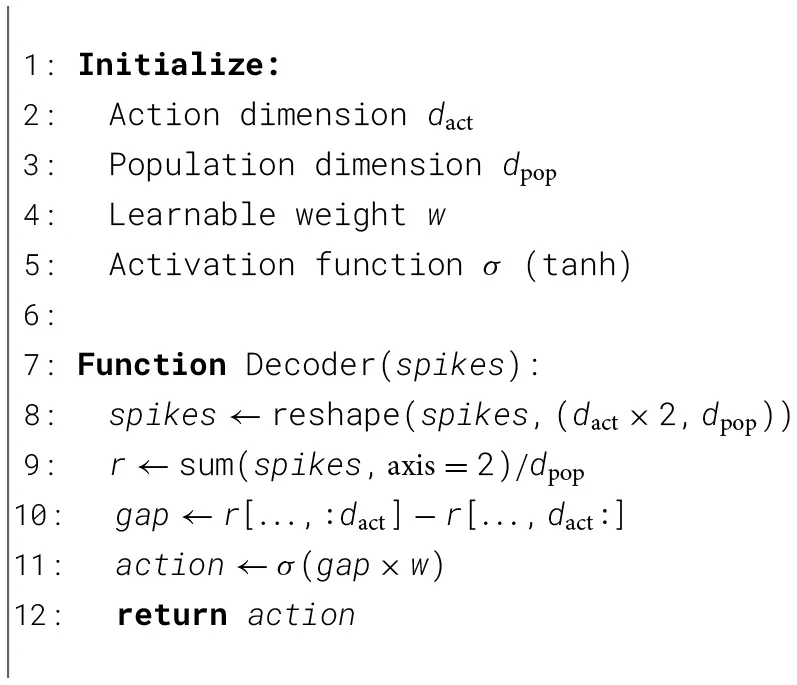

#### 3.3.3 Critic network

While a standard TD3 critic network estimates the Q-function directly from the raw state *s* and action *a*, the proposed critic network additionally incorporates the state embedding *z*_*s*_ and the state-action embedding *z*_*sa*_. The state-action pair (*s, a*) is first processed through a linear layer to extract intermediate features, which are then concatenated with *z*_*s*_ and *z*_*sa*_. The raw inputs preserve complete information, whereas the learned embeddings capture the environmental transition dynamics. However, introducing embeddings increases the input dimensionality of the Q-function, which can lead to extrapolation errors (Fujimoto et al., 2019). Following prior research, we mitigate this issue by applying value clipping to the target Q-values, constraining them within the range of Q-values observed during training (Fujimoto et al., 2023). The critics are trained by minimizing the Huber loss between their Q-value estimates and the temporal-difference targets. These estimates are then used to update the actor to increase the estimated action value. During the actor update, gradients are propagated from the critic's evaluation to the actor, while the critic and SALE encoder parameters are held fixed. Consequently, the learned embeddings participate in policy learning through both the actor's input representation and the critic's evaluation of actions. Because the critic network is only used during training and is not required for inference, it is implemented using an ANN.

#### 3.3.4 Replay buffer

A standard replay buffer *D* stores the state *s*, action *a*, reward *r*, next state *s*′, and done signal *d*. In this study, we additionally store the membrane potentials *m, m*′, representing the internal state of the spiking actor network. This allows for the reproduction of the internal state at the time of each transition, enabling accurate action inference under identical conditions. For experience replay, we adopt loss-adjusted prioritized experience replay (LAP) (Fujimoto et al., 2020), which modifies prioritized experience replay (Schaul et al., 2016) based on the relationship between non-uniform sampling and the loss function. By introducing a lower bound on sampling priorities, LAP suppresses outlier bias and "dead" transitions, facilitating more stable learning. The sampling priority for transition *i* is defined based on the TD error δ_*i*_ as


$$\begin{array}{l} p\left(i\right)=\max\left(|\delta_{i}{|}^{\alpha },1\right), \end{array}$$ (3)

where α controls the degree of prioritization. The corresponding sampling probability is given by


$$\begin{array}{l} P\left(i\right)=\frac{p\left(i\right)}{\sum_{j\in D}p\left(j\right)}. \end{array}$$ (4)

## 4 Experiments and results

This section describes experiments conducted on multiple continuous control tasks to verify the effectiveness of the proposed method. First, we compared the proposed method with PopSAN (Tang et al., 2021) and MDC-SAN (Zhang et al., 2022), representative SNN-based DRL methods that combine spiking actor networks with deep critic networks. Next, we performed an ablation study to clarify the contribution of the embeddings to performance. Additionally, we investigated how variations in embedding dimensionality influence learning performance. Finally, we estimated and evaluated the energy consumption during inference.

For our performance benchmarks, we used continuous control tasks on the MuJoCo simulator (Todorov et al., 2012) provided by OpenAI Gym (Brockman et al., 2016). The environments selected for evaluation were Ant-v4, HalfCheetah-v4, Walker2d-v4, and Hopper-v4. A brief overview of these tasks is provided below.

- **Ant-v4**: A 3D quadruped robot in which the agent must coordinate four legs to move forward. The observation and action spaces have 27 and 8 dimensions, respectively.
- **HalfCheetah-v4**: A planar cheetah-like robot tasked with running forward by applying torques to its joints. The observation and action spaces have 17 and 6 dimensions, respectively.
- **Walker2d-v4**: A 2D bipedal walker that must learn efficient forward locomotion. The observation and action spaces have 17 and 6 dimensions, respectively.
- **Hopper-v4**: A 2D one-legged hopping robot tasked with forward hopping while maintaining balance. The observation and action spaces have 11 and 3 dimensions, respectively.

For each task, training was performed over one million steps, and performance was evaluated by computing the average cumulative reward over 10 episodes at fixed intervals. Each method was evaluated using 10 random seeds, and the averaged results are reported. The networks were implemented using PyTorch (Paszke et al., 2019) and snnTorch (Eshraghian et al., 2023).

The actor network is implemented as an FC-SNN with two hidden layers of 256 neurons each and an output layer, all using LIF neurons. The critic network uses a fully connected architecture with a hidden layer of 256 neurons and ELU activation. Both networks were optimized using the Adam (Kingma and Ba, 2017) with a learning rate of 3 × 10^−4^. The discount factor was set to 0.99. Gaussian exploration noise with a standard deviation of 0.1 was added to the actor output during training. In addition, Gaussian noise with a standard deviation of 0.2 was applied to the target policy for smoothing. The replay buffer capacity was set to 1 × 10^6^, and the batch size was set to 256. The initial random exploration phase was set to 25,000 steps, and the target networks were updated every 250 steps.

### 4.1 Comparative evaluation with baselines

We evaluated the performance of the proposed method by comparing it with the PopSAN and MDC-SAN baselines. Figure 3 illustrates the learning curves over one million time steps, whereas Table 1 summarizes the maximum average rewards achieved by each method. On Ant-v4, HalfCheetah-v4, and Walker2d-v4, the proposed method outperformed both baselines from the early stages of learning and maintained these performance gaps as training progressed. As summarized in Table 1, the proposed method achieved higher maximum average rewards than both baselines on these three tasks.

**Figure 3:**
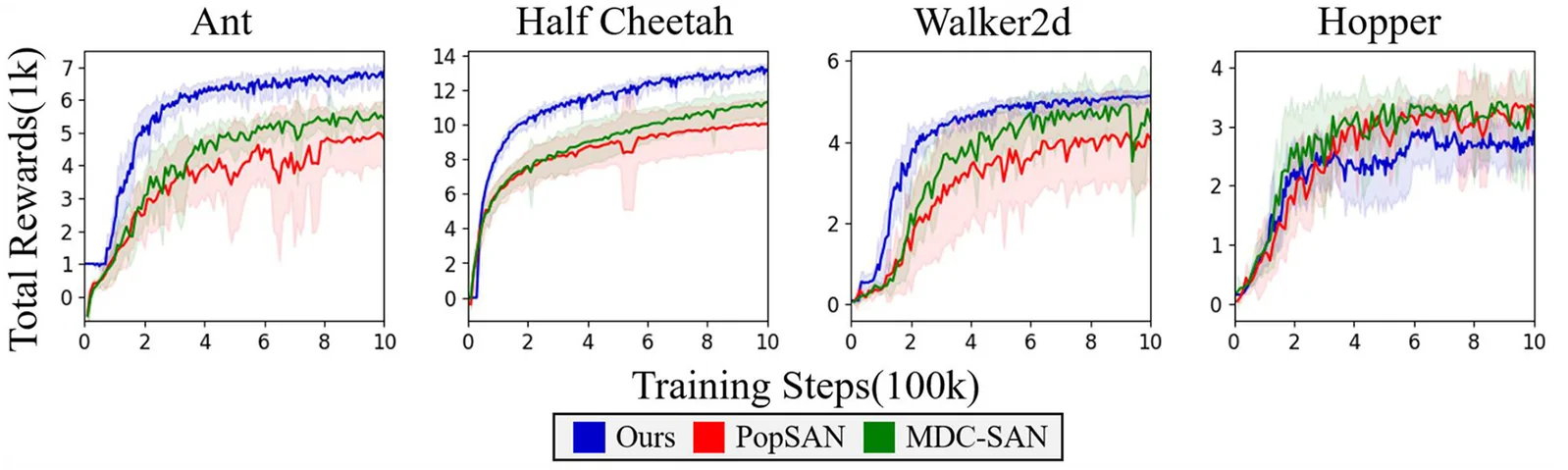
Learning curves comparing the performance of the proposed method with the PopSAN and MDC-SAN baselines across MuJoCo benchmark tasks. Results are averaged over 10 random seeds, and the shaded regions represent the standard deviation.

**Table 1:** Maximum average rewards achieved on MuJoCo benchmark tasks.

Method	Setting	Ant-v4	HalfCheetah-v4	Walker2d-v4	Hopper-v4
Ours	*d*_*z*_*s*__, *d*_*z*_*sa*__ = 256	6901 ± 395	13133 ± 250	5043 ± 731	3341 ± 148
	*d*_*z*_*s*__, *d*_*z*_*sa*__ = 128	6954 ± 232	13229 ± 312	5492 ± 246	3282 ± 178
	*d*_*z*_*s*__, *d*_*z*_*sa*__ = 64	6933 ± 91	13405 ± 190	5166 ± 154	3445 ± 98
	*d*_*z*_*s*__, *d*_*z*_*sa*__ = 32	6243 ± 835	12757 ± 268	4742 ± 721	3380 ± 152
	*d*_*z*_*s*__, *d*_*z*_*sa*__ = 16	6090 ± 754	12156 ± 240	4649 ± 337	3428 ± 62
PopSAN	–	5235 ± 912	10209 ± 1433	4456 ± 944	3440 ± 111
MDC-SAN	–	5811 ± 157	11367 ± 770	5174 ± 637	3459 ± 128
w/o Embedding	–	5690 ± 66	7245 ± 293	3648 ± 295	3178 ± 102
w/o Policy Embedding	–	6067 ± 583	8968 ± 245	4839 ± 509	3276 ± 145
w/o Critic Embedding	–	5804 ± 625	7765 ± 861	4191 ± 400	3241 ± 130

Results are averaged over 10 random seeds and reported as mean ± standard deviation. The best-performing result for each task is highlighted in blue. Any result that is not statistically significantly different from the best-performing result is highlighted in orange, according to Welch's *t*-test at a significance level of 0.05.

### 4.2 Ablation study on network components

We conducted an ablation study to examine the contribution of embeddings to the spiking actor and critic networks. First, we compared the proposed method with a configuration without embeddings, in which the actor receives only the raw state *s*, while the critics receive only the raw state *s* and action *a*. Figure 4 shows that the proposed method achieved higher rewards from the early stages of learning on Ant-v4, HalfCheetah-v4, and Walker2d-v4, and maintained these performance gaps as training progressed. As summarized in Table 1, the proposed method also achieved higher maximum average rewards on all four tasks, although the improvement on Hopper-v4 was relatively small. These results support the effectiveness of introducing embeddings into the SNN-based DRL framework, while also indicating that the magnitude of the improvement varies across tasks.

**Figure 4:**
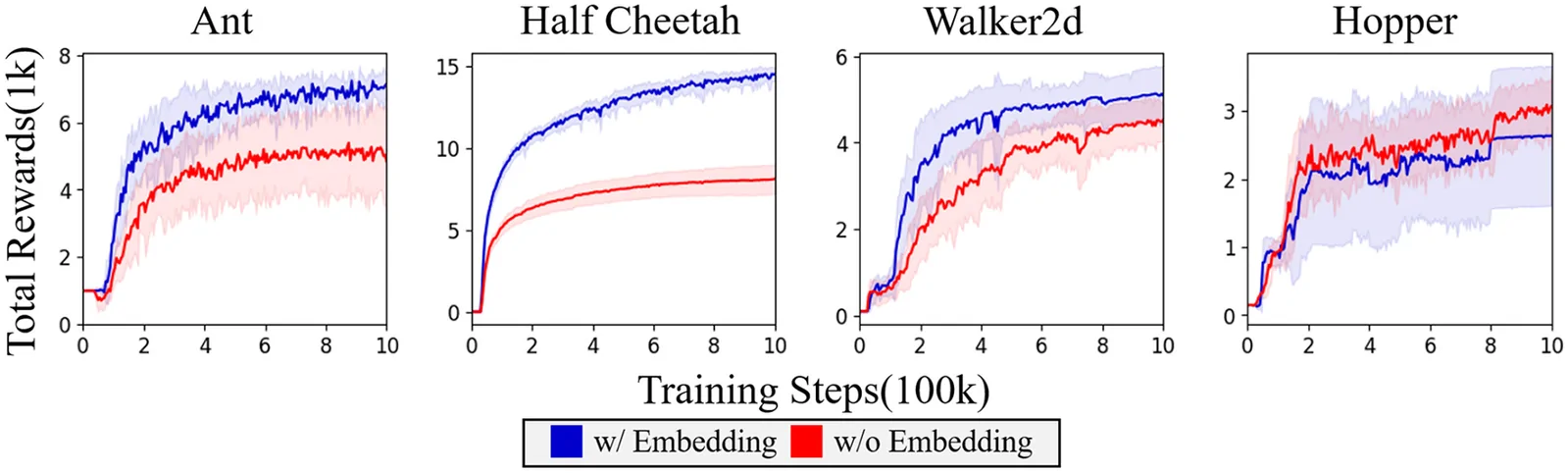
Learning curves evaluating the proposed method with and without embeddings in both the actor and critic networks on MuJoCo benchmark tasks. “w/ Embedding” and “w/o Embedding” indicate the presence and absence of embeddings in both the actor and critic, respectively. Results are averaged over 10 random seeds, and the shaded regions represent the standard deviation.

Next, we examined the contribution of the state embedding *z*_*s*_ to the spiking actor network by comparing the proposed method with a configuration in which the actor receives only the raw state *s*. The critics use embeddings in both configurations. While Fujimoto et al. demonstrated the effectiveness of providing state embeddings to ANN-based actors, this comparison examines whether this benefit extends to a spiking actor, in which the embeddings are converted into spike trains. As shown in Figure 5, providing *z*_*s*_ to the spiking actor resulted in higher rewards as learning progressed on Ant-v4, HalfCheetah-v4, and Walker2d-v4. These results support the effectiveness of directly providing the state embedding to the spiking actor, even when the critics already use embeddings.

**Figure 5:**
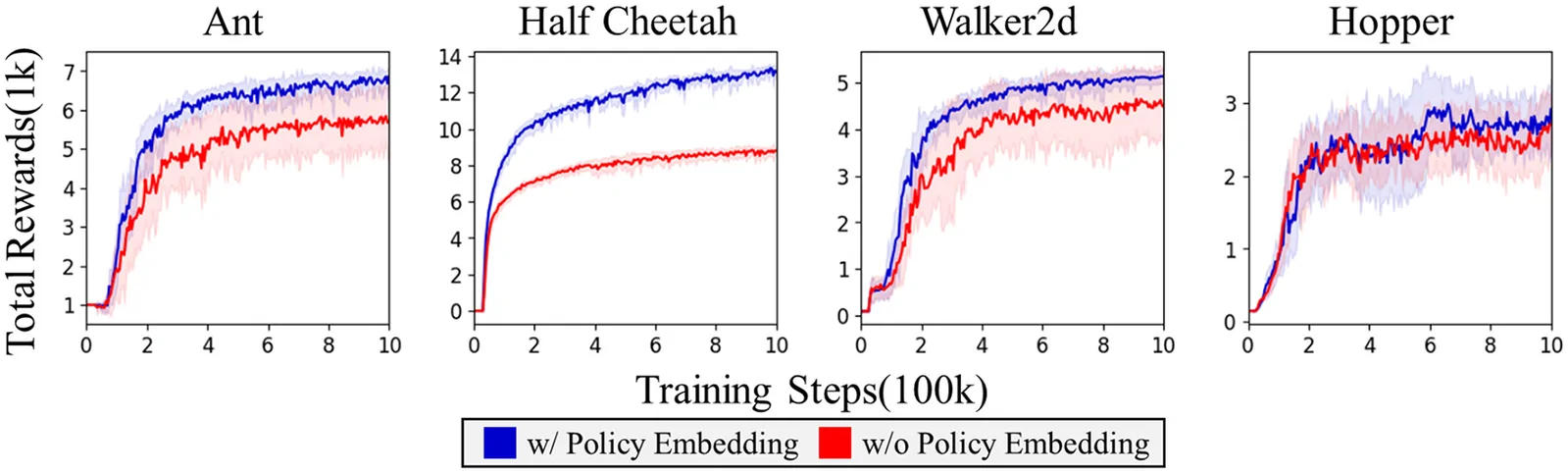
Learning curves comparing the proposed method with and without the state embedding *z*_*s*_ in the actor network across MuJoCo benchmark tasks. “w/ Policy Embedding” and “w/o Policy Embedding” denote the presence and absence of *z*_*s*_ in the actor, respectively. Results are averaged over 10 random seeds, and the shaded regions represent the standard deviation.

Finally, we examined the contribution of embeddings to the critic networks by comparing the proposed method with NoCriticEmb, in which both the state embedding *z*_*s*_ and the state–action embedding *z*_*sa*_ are removed from the critics. The actor receives the state embedding in both configurations. As shown in Figure 6, the proposed method achieved higher rewards over most of the training process on Ant-v4, HalfCheetah-v4, and Walker2d-v4, whereas the learning curves largely overlapped on Hopper-v4. As summarized in Table 1, the proposed method achieved higher maximum average rewards on all four tasks, although the improvement on Hopper-v4 was relatively small. Together with the preceding comparison in which the state embedding was removed from the actor, these results support the effectiveness of providing embeddings to both the actor and critics under the evaluated conditions.

**Figure 6:**
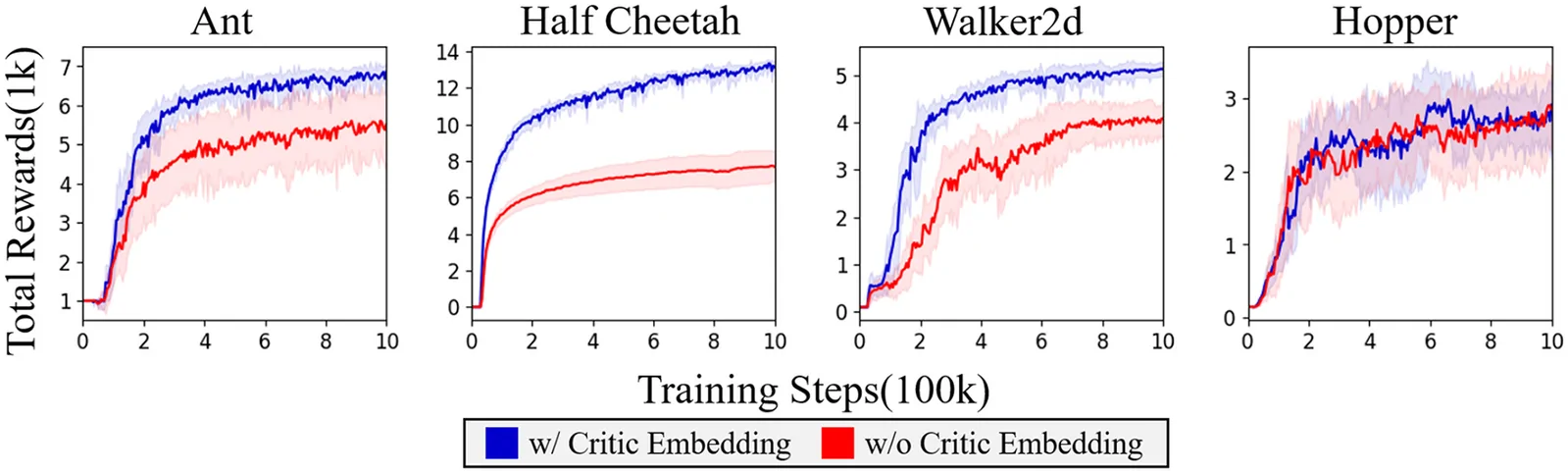
Learning curves comparing the proposed method with and without embeddings in the critic networks across MuJoCo benchmark tasks. “w/ Critic Embedding” and “w/o Critic Embedding” denote the presence and absence of the state embedding *z*_*s*_ and state–action embedding *z*_*sa*_ in the critics, respectively. The actor receives the state embedding *z*_*s*_ in both configurations. Results are averaged over 10 random seeds, and the shaded regions represent the standard deviation.

### 4.3 Investigation of embedding dimensionality

To clarify how input dimensionality affects the learning performance of the spiking actor network, we investigated the impact of embedding dimensionality. In addition to the default 64-D setting, we compared configurations with 16, 32, 128, and 256 dimensions, using the same dimensionality for the state embedding *z*_*s*_ and the state–action embedding *z*_*sa*_. Figure 7 presents the corresponding learning curves, while Table 1 summarizes the maximum average rewards for each embedding dimensionality. Across the evaluated tasks, the highest mean maximum average rewards were achieved with either 64 or 128 dimensions, and increasing the dimensionality to 256 did not yield further performance improvements. On Hopper-v4, however, the 16-D configuration achieved a mean maximum average reward close to that of the 64-D configuration. The standard deviation of the maximum average reward was lowest at 64 dimensions on Ant-v4, HalfCheetah-v4, and Walker2d-v4, whereas it was lowest at 16 dimensions on Hopper-v4.

**Figure 7:**
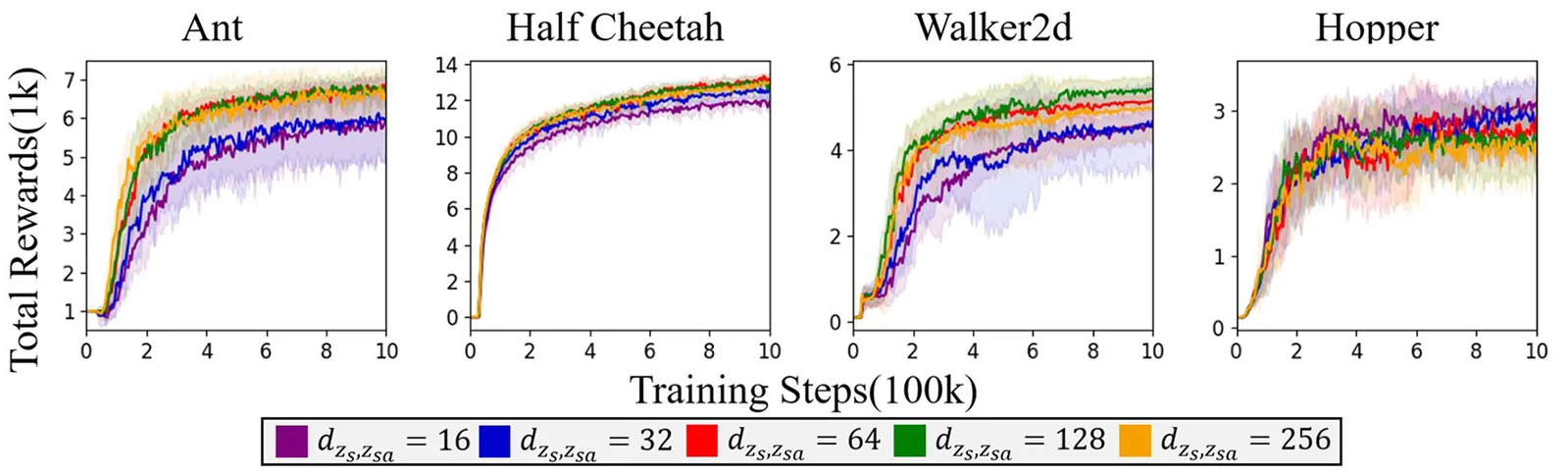
Learning curves comparing the effect of different embedding dimensions (*d*_*z*_*s*__, *d*_*z*_*sa*__ = 16, 32, 64, 128, 256) in the proposed method on MuJoCo benchmark tasks. Results are averaged over 10 random seeds, and the shaded regions represent the standard deviation.

### 4.4 Assessment of energy consumption

Since the primary motivation for using SNNs is their high energy efficiency on neuromorphic devices, this study estimates the energy consumption during inference. The baseline for comparison is a conventional ANN-based TD7 model (Fujimoto et al., 2023). The hardware assumed for deployment was determined based on prior work (Xu et al., 2026). For the ANN-based TD7 model, energy consumption was estimated assuming deployment on modern NPUs (Millar et al., 2025). On this hardware, an energy cost of 3.97pJ per multiply-accumulate operation (MAC) has been reported (Millar et al., 2025). In contrast, for the FC-SNNs in the proposed method, energy consumption was estimated assuming execution on ROLLS (Qiao et al., 2015), a neuromorphic chip. On this neuromorphic chip, an energy consumption of 77fJ per synaptic operation (SOP) has been reported (Indiveri et al., 2015).

In this evaluation, one inference corresponds to one action decision in the environment. For both models, the evaluation included the fully connected layers of the State Encoder and actor networks used during inference. The total MAC and SOP counts per inference were calculated separately across these layers. The energy consumption per inference was then estimated by multiplying these totals by the corresponding reference energy costs per operation and summing the results. For a fully connected layer, the required number of operations per inference is *D*_in_*D*_out_ MACs for ANNs and *fD*_in_*D*_out_ SOPs for SNNs. Here, *D*_in_ and *D*_out_ are the input and output dimensions, respectively and *f* is the average number of spikes per input neuron per internal time step, expressed as a fraction rather than a percentage. The State Encoder is an ANN-based fully connected network, requiring 256(*N*+256+*d*_*z*_*s*__) MACs per inference, where *N* is the observation dimension of each task. Its energy consumption is estimated by multiplying this MAC count by the reference energy cost of 3.97pJ per MAC, which is the same value used for the ANN-based TD7 model.

For the FC-SNN, we use (*D*_in_, *D*_out_) to denote a fully connected layer with input dimension *D*_in_ and output dimension *D*_out_. The FC-SNN in the spiking actor network consists of three fully connected layers: FC1 (2(*N*+*d*_*z*_*s*__)*d*_pop_, 256), FC2 (256, 256), and FC3 (256, 2*Md*_pop_), where *M* is the action dimension, and *d*_pop_ is the population dimension. The factor of two arises from the use of separate positive and negative populations. Table 2 reports the average firing rates of the input spike trains and the LIF neurons in each layer, obtained by running each trained model for one episode. As shown in Table 3, the proposed method had lower estimated energy consumption per inference than the ANN-based TD7 model across all evaluated tasks under the adopted reference energy costs.

**Table 2:** Average firing rates (%) of the input spike trains and the outputs of FC1, FC2, and FC3 in the proposed method.

Task	Input Spikes	FC1	FC2	FC3
Ant-v4	11.6	18.7	14.9	9.1
HalfCheetah-v4	15.7	22.6	16.7	36.5
Walker2d-v4	10.4	9.0	7.2	22.7
Hopper-v4	9.0	5.0	4.0	21.4

**Table 3:** Energy consumption per inference for different MuJoCo benchmark tasks.

Method	Ant-v4	HalfCheetah-v4	Walker2d-v4	Hopper-v4
Ours	368.5	360.9	354.1	345.4
ANN (TD7)	1363.9	1341.5	1341.5	1326.3

The energy unit is nanojoules (nJ).

## 5 Discussion

Across multiple tasks, the proposed method demonstrated superior performance during the early stages of learning and achieved higher final performance in most tasks. This improvement is primarily attributed to the introduction of SALE, which provides embeddings learned from the transition dynamics of the environment. Because the Q-function estimates expected future rewards, accurately capturing these transition dynamics is essential for reliable action-value estimation. SALE represents these transition dynamics within a latent space and extracts meaningful features that inform the action value estimation process. By leveraging such embeddings, the critic can estimate the Q-function based on latent representations that capture transition dynamics and task-relevant information from the onset of learning. Consequently, policy updates become more stable and efficient. Furthermore, the role of the state embedding *z*_*s*_ in the proposed method is not limited to assisting Q-function estimation in the critic. In SNNs, information is propagated through spike trains rather than continuous-valued activations. Therefore, compared with an ANN-based actor, a spiking actor network is more constrained in forming continuous-valued features that are useful for policy learning within the network. For this reason, providing the spiking actor network with state embeddings *z*_*s*_ learned from environmental transition dynamics provides an important input representation. This enables the spiking actor network to learn a policy not only from spike-based representations of the raw state but also from auxiliary representations that reflect transition dynamics.

The ablation results further supported this interpretation. As shown in Figure 4 and Table 1, removing the embeddings from both the actor and critic networks reduced the maximum average rewards across the evaluated tasks, supporting the overall contribution of SALE to the proposed framework. Furthermore, as shown in Figure 5, when the state embedding *z*_*s*_ input to the spiking actor network was removed while the critic still used embeddings, performance decreased across multiple tasks. This result supports the effectiveness of directly providing the state embedding *z*_*s*_ to the spiking actor network as an auxiliary representation for policy learning. As shown in Figure 6 and Table 1, removing the embeddings from the critic networks while retaining the state embedding in the actor also reduced performance, particularly on Ant-v4, HalfCheetah-v4, and Walker2d-v4. This indicates that providing embeddings only to the actor does not reproduce the performance achieved when both networks use them. These results are consistent with transition-aware features supporting action-value estimation in the critic and, consequently, the policy updates guided by its evaluations. Together, the ablation results support the use of embeddings through both the actor's input representation and the critic's evaluation of actions. As shown in Table 1, on Hopper-v4, the proposed method achieved a higher mean maximum average reward than the ablated configurations, although the improvements were smaller than on the other tasks. Its relatively constrained hopping pattern may allow the raw inputs to capture much of the transition information needed for action evaluation and policy learning, reducing the additional benefit of the learned embeddings. These results suggest that the effectiveness of the proposed method depends on the complexity of the transition dynamics and state–action interactions in each task.

As shown in Figure 8 and Table 1, the optimal embedding dimensionality differs across tasks. The optimal dimensionality appears to depend not simply on the size of the task's observation space, but rather by the complexity of the underlying dynamical system. As shown in Table 1, on Hopper-v4, the 16-D configuration achieved a maximum average reward close to that of the 64-D configuration. Its relatively constrained one-legged hopping pattern may allow the relevant transition features to be represented with fewer embedding dimensions. On Ant-v4, increasing the embedding dimensionality from 16 to 64 improved the maximum average reward, as shown in Figure 8 and Table 1. This is consistent with the interpretation that coordinating multiple legs benefits from a richer representation of state–action interactions. However, increasing dimensionality beyond a certain range did not provide further benefits. As shown in Figure 8 and Table 1, performance largely saturated beyond 64 dimensions on Ant-v4, while HalfCheetah-v4 achieved its highest mean maximum average reward with 64 dimensions. Walker2d-v4 benefited from increasing the embedding dimensionality to 128, but performance declined when the dimensionality was further increased to 256. These results suggest that the useful range of embedding dimensions varies across tasks. The saturation and task-dependent decline in performance at larger embedding dimensions may be related to the characteristics of spike-based computation. Since the state embedding *z*_*s*_ is converted into spike trains, increasing its dimensionality can increase the spike activity processed by the spiking actor network. This may complicate the integration of task-relevant features and make policy learning less stable, particularly when additional dimensions provide limited useful information. Thus, although larger embeddings offer greater representational capacity, their benefits may be offset by the increased complexity of processing spike-based inputs. Therefore, embedding dimensionality should be selected by considering both the transition features required by the task and the ability of the spiking actor network to use these features effectively.

**Figure 8:**
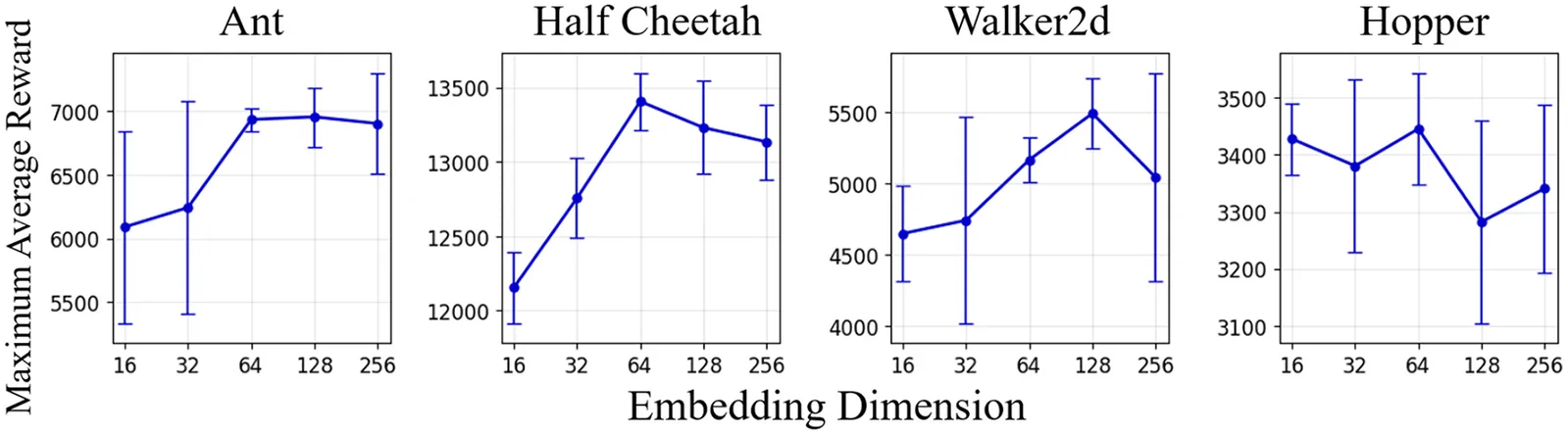
Maximum average rewards across embedding dimensions on MuJoCo benchmark tasks. Results are averaged over 10 random seeds, and the error bars represent the standard deviation.

As shown in Table 3, the proposed method exhibited lower inference energy consumption than the ANN-based DRL model. This result suggests that using a spiking actor network enables decision-making in continuous control tasks to be performed with lower energy consumption. The ability to improve learning performance and sample efficiency through transition-aware embeddings while reducing the estimated inference energy consumption is important for robotic control under limited computational resources and power capacity. Indeed, SNN-based DRL has been applied to mapless navigation using neuromorphic hardware (Tang et al., 2020) and force-control tasks in industrial robots (Amaya et al., 2024), where its real-world operation and energy efficiency have been evaluated. Therefore, these results indicate one direction for extending SNN-based DRL to resource-constrained embodied AI systems, including robotic control systems, by improving learning performance and sample efficiency while suppressing inference-time energy consumption.

## 6 Conclusion

This paper proposed a method incorporating transition-aware embeddings into both the spiking actor network and critic networks to improve performance and sample efficiency in SNN-based DRL. The proposed method was evaluated on four continuous control tasks from OpenAI Gym. It outperformed the baseline methods during the early stages of learning on most tasks and achieved higher final performance across several tasks. Furthermore, in SNN-based DRL, appropriately setting the embedding dimensionality is important for achieving stable learning, as it influences the balance between representational capacity and stability. The energy-consumption analysis also indicated lower estimated inference energy consumption than that of the ANN-based DRL model.

Future work should validate the proposed method on real robotic platforms and neuromorphic hardware, including measurements of actual power consumption and evaluations of robustness in real-world environments.

## Data Availability

The raw data supporting the conclusions of this article will be made available by the authors, without undue reservation.
